# Perceived return-to-work pressure following cardiovascular disease is associated with age, sex, and diagnosis: a nationwide combined survey- and register-based cohort study

**DOI:** 10.1186/s12889-022-13494-1

**Published:** 2022-05-27

**Authors:** Sidsel Marie Bernt Jørgensen, Nina Føns Johnsen, Thomas Alexander Gerds, Stig Brøndum, Thomas Maribo, Gunnar Gislason, Maria Kristiansen

**Affiliations:** 1 Danish Heart Foundation, Section of Cardiovascular Research, Vognmagergade 7, 3rd Floor, 1120 Copenhagen K, Denmark; 2 Department of Public Health & Center for Healthy Aging, University of Copenhagen, Copenhagen, Denmark; 3 Department of Public Health, Section of Biostatistics, University of Copenhagen, Copenhagen, Denmark; 4 Department of Public Health, Aarhus University, Aarhus, Denmark; 5 DEFACTUM, Central Denmark Region, Aarhus, Denmark; 6 Department of Cardiology, Copenhagen University Hospital Herlev and Gentofte, Hellerup, Denmark; 7 Department of Clinical Medicine, University of Copenhagen, Copenhagen, Denmark

**Keywords:** Cardiac rehabilitation, Cardiovascular disease, Return to work, Employment, Vocational counselling, Vocational rehabilitation

## Abstract

**Background:**

Return to work is a key rehabilitation goal for people with cardiovascular disease (CVD) because employment matters to individuals and societies. However, people recovering from CVD often struggle with returning to work and maintaining employment. To identify people in need of vocational counselling, we examined the probability of feeling under pressure to return to work following CVD.

**Methods:**

We conducted a combined survey- and register-based study in a randomly selected, population-based cohort of 10,000 people diagnosed with atrial fibrillation, heart failure, heart valve disease, or ischaemic heart disease in 2018. The questionnaire covered return-to-work items, and we reported the probabilities of feeling under pressure to return to work with 95% confidence intervals (CIs) in categories defined by sex, age, and CVD diagnosis.

**Results:**

The survey response rate was 51.1%. In this study, we included 842 respondents (79.7% men) aged 32–85 years, who had returned to work following a sick leave. Overall, 249 (29.7%) had felt pressure to return to work. The probability of feeling under pressure to return to work ranged from 18.3% (95% CI: 13.1–24.6) among men aged > 55 years with atrial fibrillation to 51.7% (95% CI: 32.5–70.6) among women aged ≤ 55 years with atrial fibrillation. In addition, 66.0% of all respondents had not been offered vocational rehabilitation, and 48.6% of those who reported a need for vocational counselling had unmet needs. Survey responses also indicated that many respondents had returned to work before feeling mentally and physically ready.

**Conclusion:**

A substantial proportion of people with cardiovascular disease feel under pressure to return to work, and this pressure is associated with age, sex, and diagnosis. The results show that vocational rehabilitation must be improved and emphasize the importance of ensuring that cardiac rehabilitation programmes include all core rehabilitation components.

## Introduction

Despite improved survival, cardiovascular disease (CVD) remains a leading cause of mortality and increased disease burden in Europe and is increasingly common in the working-age population [1, 2]. In Denmark, more than 56,000 people are diagnosed with CVD each year [3], and according to The Danish Heart Statistics approximately 36% of these are between 35 and 65 years old [3, 4]. Hence, a great proportion of people living with CVD is of working-age, and for these people facilitating return to work is a key rehabilitation goal, because employment is highly valued by individuals and societies [5–7].

Cardiac rehabilitation may facilitate return to work [8, 9], and international recommendations include vocational counselling as part of comprehensive rehabilitation programmes [10]. However, most European countries do not have clear guidelines [9], and more emphasis on vocational rehabilitation is needed [9, 11]. The aim of cardiac rehabilitation is to improve health and quality of life and allow people, as far as possible, to return to their activities of daily living [12], and for employed people with CVD work resumption is an important aspect of returning to normality [13–16]. Return to work is associated with better psychosocial well-being and health related quality of life [16], and is of great importance for social relationships, income and purpose in life [15]. Return to work is a complex process shaped by a range of factors including personal characteristics, the severity of the disease, work-related factors (e.g., job position and working conditions), national compensation policies, and the structure of the healthcare system (e.g., access to rehabilitation programmes and vocational counselling) [17, 18]. Previous studies have demonstrated that CVD affects the ability to work and that return-to-work rates are moderate with variation across countries and patient groups [8, 9, 18–22]. Risk factors associated with failure to return to work after CVD include female sex [19–21, 23], older age [21, 23], severity of the disease [20, 24], comorbidities [19–21, 23], lower educational level [19–21], and low income [19, 21]. Additionally, type of employment and occupational requirements, such as white or blue-collar work [8, 9], night shifts, and the ability to commute shape return-to-work [9]. Researchers have also documented that many people with CVD struggle with sustaining employment after initial return to work [19, 20, 22], have difficulties with returning to work at the pre-CVD level [13], and experience a number of barriers for returning to work, such as physical and mental incapacity, co-morbidities, unfavorable terms of employment, and motivational problems [13].

Struggles with returning to work and sustaining employment may be due to insufficient vocational rehabilitation, which results in patients feeling compelled to return to work while they are still recovering. To support people who are returning to work, help them continue their employment, and provide cardiac rehabilitation, we need to identify those in need of vocational rehabilitation. To improve our understanding of the return-to-work experience of people with CVD, we conducted a nationwide survey and linked the responses with register data. We investigated the likelihood of patients feeling under pressure to return to work and investigated whether this was influenced by sex, age, or CVD diagnosis. The results provide valuable insights, which may be used to improve targeted vocational rehabilitation and facilitate return to work after CVD.

## Methods

### Study design and population

We conducted a combined survey- and register-based cohort study: data collected from a nationwide population-based survey were linked to data from several nationwide administrative registries. In Denmark, all residents are assigned a unique and permanent personal identification number, which is available in all national registries and allows accurate data to be assigned to each individual [25]. The Danish National Patient Register contains information on all hospital admissions, in accordance with the tenth revision of the International Classification of Diseases (ICD-10) codes [26]. The patients in this study were selected from the Danish National Patient Register and formed a random population-based cohort of 10,000 people who had a hospital admission (in- or outpatient contact) in 2018 with a discharge diagnosis of atrial fibrillation (AF) (ICD-10: I48), heart failure (HF) (ICD-10: I11.0, I13.0, I13.2, I50, I42.0, I42.6, I42.7, I42.9), heart valve disease (HVD) (ICD-10: I05–I08, I34–I37) or ischaemic heart disease (IHD) (ICD-10: I20–I25). Because of the overall low incidence of CVD in younger individuals [2], and because we wanted to include people of working-age with a foothold on the labour market, patients were included if they were ≥ 35 years of age. In addition, survey participants were residents in Denmark and alive when the cohort was established in August 2020. In this study, we included respondents who reported being employed (including self-employment, part-time employment etc.) at the time of diagnosis and who had returned to work following a sick leave due to their diagnosis in 2018.

### Questionnaire development and distribution

The Life With Heart Disease study was established in 2013 to examine heart patients’ experiences with the Danish healthcare system and to assess these patients’ physical and mental health status. The first survey was conducted in 2014 [27], and the second survey was conducted between October 2020 and February 2021. This study was based on the latter survey. The questionnaire was developed in collaboration with experts and healthcare professionals with professional experience of CVD (e.g., social workers, nurses, psychologists, and cardiac rehabilitation experts), as well as on similar surveys. The survey was pilot tested using cognitive interviewing techniques to ensure that respondents understood the questions and were able to use the response options effectively. The final questionnaire consisted of 51 questions across 11 themes: treatment and experiences at the hospital; contact with health professionals; information; relatives; medication; social and economic aspects; everyday life; health and wellbeing; the coronavirus disease 2019 (COVID-19) pandemic; rehabilitation and support; and work life and return to work. Survey items from the latter two themes were included in this study. The questionnaire included both open and closed questions, as well as internationally validated scales (e.g., the 5-item World Health Organization Well-Being Index and the Hospital Anxiety and Depression Scale). The survey was either sent to patients’ digital mailboxes (80%) or posted to patients’ home addresses including a prepaid envelope if patients were exempt from receiving digital post (20%). Two reminders were sent to patients’ digital mailboxes or home addresses, and the remaining non-respondents were contacted by telephone.

### Measures and data sources

Data from The Life With Heart Disease study were linked to data from nationwide administrative registries obtained from Statistics Denmark. The Danish registries are validated and have been described in detail previously [25, 26, 28–30].

#### Register data

Information on CVD type was obtained from The Danish National Patient Register [26]. Information regarding sex, age at diagnosis, country of birth, cohabitation, and place of residence was obtained from The Danish Civil Registration System [25]. Information regarding educational level was obtained from the Population Education Register [28], and information regarding household income was obtained from the Income Statistics Register [29].

#### Survey data

The outcome of interest, pressure to return to work, was measured using one item: “Have you felt under pressure to return to work after your sick leave?”. The response options covered seven possible sources of pressure: 1) Yes, I have felt under pressure by the job counselling centre; 2) Yes, I have felt under pressure by my employer; 3) Yes, I have felt under pressure by my colleagues; 4) Yes, I have felt under pressure by my relatives; 5) Yes, I have felt a financial pressure; 6) Yes, I have felt under pressure for other reasons; 7) No, I have not felt under pressure. The respondent was able to select one or more options, and the item was dichotomized into ‘No’ pressure (score = 7) or ‘Yes’ (score = 1–6) if the respondent had felt under pressure to return to work.

Additional survey items on return to work and vocational rehabilitation were included. Offers of vocational rehabilitation were collected using one item that covered all core components of cardiac rehabilitation: “Were you offered counselling regarding employment and return to work in relation to your heart disease, either at the hospital, from your general practitioner, or in your municipality?”. The response options were: 1) Yes, and I accepted; 2) Yes, I accepted, but dropped out; 3) Yes, but I did not accept; 4) No; 5) Do not know/Not relevant. Scores 1–3 correspond to having been offered vocational rehabilitation.

The need for professional vocational counselling and the unmet need for professional vocational counselling were measured using one item: “Did you experience a need for professional vocational counselling, e.g., from a social worker, union, job consultant etc.?” The response options were: 1) Yes, and I received professional counselling; 2) Yes, but I did not receive professional counselling; 3) No. To measure the need for professional vocational counselling the item was dichotomized into ‘Yes’ (score = 1–2) or ‘No’ (score = 3). To measure the unmet need for vocational counselling the item was dichotomized into ‘Yes’ (score = 2) or ‘No’ (score = 1, 3).

Employer support in the return-to-work process was measured using one item: “Has your employer helped you maintain your employment?”. The response options were: 1) Not at all; 2) Very little; 3) Yes, somewhat; 4) Yes, to a great extent; 5) Don’t know. Scores 1–2 correspond to no support, and scores 3–4 correspond to having received employer support.

Reasons to return to work were measured using one item: “What were the reasons you returned to work at the time you did?”. The response options covered eight reasons, including “I felt physically ready to return to work” and “I felt mentally ready to return to work”.

Finally, information on comorbidities was obtained using one item covering 14 illnesses, including cancer, diabetes, anxiety, and depression. This item was dichotomized into ‘Yes’ and ‘No’.

### Statistical analysis

The probability of feeling under pressure to return to work was calculated as a relative frequency and reported with exact binomial 95% confidence intervals (CIs) in categories defined by diagnosis (AF, IHD, HVD, or HF), sex (male or female), and age (≤ 55 years or > 55 years). Data management and statistical analyses were performed using R software (version 4.0.3 for Windows; R Development Core Team, 2020).

## Results

**Fig. 1:**
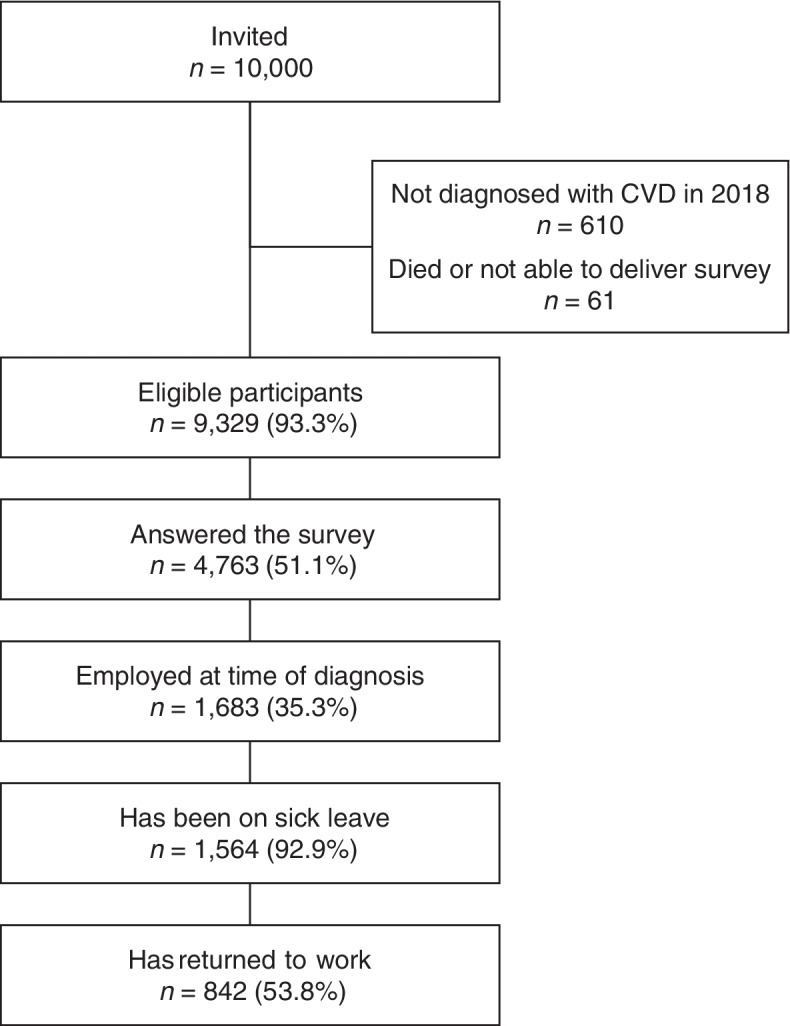
Flow chart showing the selection of the study population

Figure 1 shows the selection of the study population. The overall response rate in the Life With Heart Disease Study was 51.1%. Of the total respondents to the survey, 1,683 (35.3%) reported being in employment at the time of diagnosis; among these, 1,564 (92.9%) respondents reported having been on sick leave. Of these, 842 respondents (53.8%) had returned to work and were, therefore, included in the study population. Compared with respondents, the non-respondents were more likely to be female (53.3% vs. 47.4%, *p* < 0.001), have a higher mean age (68.6 vs. 67 years, *p* < 0.001), a lower educational level (*p* < 0.001), a lower mean income (*p* < 0.001), and have been born outside of Denmark (*p* < 0.001).

### General characteristics and aspects related to return to work

Table 1 shows the sociodemographic characteristics of the population and aspects related to return to work. The mean age was 57.4 years, most respondents were male (79.7%) and of Danish origin (94.7%). Most respondents were diagnosed with IHD (46.6%), followed by AF (43.9%), HF (20.8%) and HVD (17.2%). One-quarter of the population (25%) were diagnosed with more than one CVD (data not shown), and the majority (82.3%) had comorbidities. Approximately three-quarters of the population (77.3%) were cohabiting; approximately half of the population (51.3%) had a lower secondary education, and the mean yearly income was 49,900 EUR.

**Table 1:** Characteristics of the study population and their responses to return-to-work items (*n* = *842)*

Variable	Level	Women *n* = 171 (20.3%)	Men *n* = 671 (79.7%)	Total *n* = 842 (100%)
Age (years)	Mean (sd)	56.4 (7.5)	57.6 (7.9)	57.4 (7.8)
Age	≤ 55 years	70 (40.9%)	238 (35.5%)	308 (36.6%)
	> 55 years	101 (59.1%)	433 (64.5%)	534 (63.4%)
AF	Yes	83 (48.5%)	287 (42.8%)	370 (43.9%)
IHD	Yes	72 (42.1%)	320 (47.7%)	392 (46.6%)
HVD	Yes	21 (12.3%)	124 (18.5%)	145 (17.2%)
HF	Yes	35 (20.5%)	140 (20.9%)	175 (20.8%)
Comorbidity	Yes	90 (79.6%)	332 (83.0%)	422 (82.3%)
Country of birth	Denmark	166 (97.1%)	631 (94.0%)	797 (94.7%)
	Other	5 (2.9%)	40 (6.0%)	45 (5.3%)
Education	Basic	20 (11.8%)	113 (17.0%)	133 (15.9%)
	Secondary	81 (47.6%)	347 (52.3%)	428 (51.3%)
	Tertiary	49 (28.8%)	97 (14.6%)	146 (17.5%)
	Postgraduate	20 (11.8%)	107 (16.1%)	127 (15.2%)
Mean income (EUR)	Mean (sd)	41,600 (14,200)	52,000 (143,400)	49,900 (128,100)
Cohabitation	Alone	45 (27.3%)	136 (21.5%)	181 (22.7%)
	With others	120 (72.7%)	498 (78.5%)	618 (77.3%)
Has been offered vocational rehabilitation	No	126 (74.1%)	429 (63.9%)	555 (66.0%)
	Yes	30 (17.6%)	118 (17.6%)	148 (17.6%)
	Don’t know	14 (8.2%)	124 (18.5%)	138 (16.4%)
Employer has supported return to work	No	62 (36.5%)	227 (34.0%)	289 (34.5%)
	Yes	97 (57.1%)	365 (54.6%)	462 (55.1%)
	Don't know	11 (6.5%)	76 (11.4%)	87 (10.4%)
Need of vocational counselling	No	127 (74.3%)	564 (84.4%)	691 (82.4%)
	Yes	44 (25.7%)	104 (15.6%)	148 (17.6%)
Unmet vocational counselling needs	No	25 (56.8%)	51 (49.0%)	76 (51.4%)
	Yes	19 (43.2%)	53 (51.0%)	72 (48.6%)
Mentally ready to return to work	No	120 (74.5%)	431 (67.0%)	551 (68.5%)
	Yes	41 (25.5%)	212 (33.0%)	253 (31.5%)
Physically ready to return to work	No	96 (59.6%)	292 (45.4%)	388 (48.3%)
	Yes	65 (40.4%)	351 (54.6%)	416 (51.7%)

*Abbreviations: AF* Atrial Fibrillation, *IHD* Ischaemic Heart Disease, *HVD* Heart Valve Disease, *HF* Heart Failures. Data are presented either as means ± standard deviation (sd) or numbers (%)

Table 1 also shows that a large proportion of the population (82.4%) did not feel that they needed professional vocational counselling when returning to work. However, among those who did feel that they needed professional vocational counselling (17.6%), almost half (48.6%) reported having unmet needs. Two-thirds of the population (66%) reported that they had not been offered vocational rehabilitation after being diagnosed with CVD, and approximately one-third of the population (34.5%) reported no support from their employer during their return to work. When asked about their reasons for returning to work, approximately one-third (31.5%) and one-half (51.7%) of the population reported that they were mentally ready and physically ready to return to work, respectively. More women than men reported that they had not been offered vocational rehabilitation (74.1% vs. 63.9%), and that they had felt a need for professional vocational counselling (25.7% vs. 15.6%). In addition, fewer women than men cited mental readiness and physical readiness as their reasons for returning to work (25.5% vs. 33.0% and 40.4% vs. 54.6%, respectively).

### Feeling under pressure to return to work

**Fig. 2:**
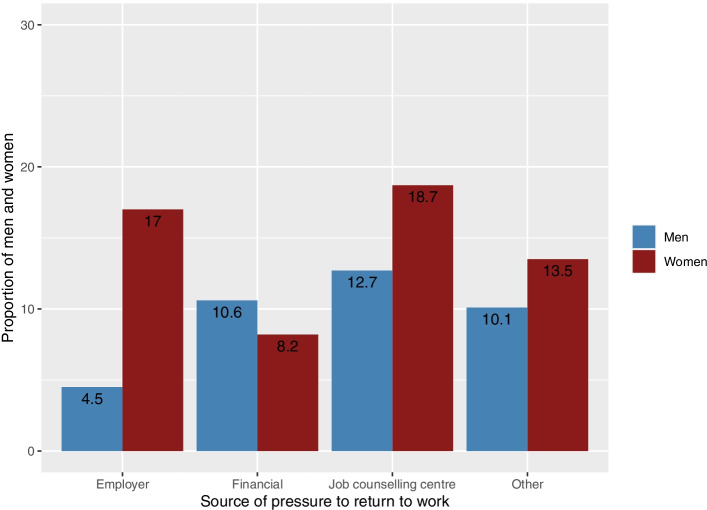
Proportion of men and women who specified particular sources of pressure to return to work. More than one answer was permitted. Pressure to return to work from colleagues or relatives is not included because these sources of pressure were reported by too few respondents

Table 2 shows that almost one-third of the study population (29.7%) had felt under pressure to return to work. The most commonly cited source of pressure to return to work was the job counselling centre (13.9% of 838 respondents). In Denmark, job counselling centres are located in municipalities, and they are responsible for the administration of sickness absence. Approximately one-tenth of respondents had felt financial pressure (10.1%) or had cited ‘other reasons’ (10.8%) for feeling pressure to return to work. Some respondents had felt pressured to return to work by their employer (7.0%), their relatives (0.6%) or their colleagues (data not shown). Figure 2 shows the proportion of men and women who specified particular sources of pressure to return to work, and for all sources, except financial, the observed proportions were higher for women than men.

**Table 2:** Prevalence of perceived pressure to return to work and the sources of this pressure (*n* = 838)

	Level	Total
Have felt under pressure to return to work	No	589 (70.3%)
	Yes	249 (29.7%)
Job counselling centre	No	725 (86.1%)
	Yes	117 (13.9%)
Employer	No	783 (93.0%)
	Yes	59 (7.0%)
Colleagues	No	
	Yes	< 5
Relatives	No	837 (99.4%)
	Yes	5 (0.6%)
Financial	No	757 (89.9%)
	Yes	85 (10.1%)
Other reason	No	750 (89.2%)
	Yes	91 (10.8%)

Data are presented as numbers (%)

**Fig. 3:**
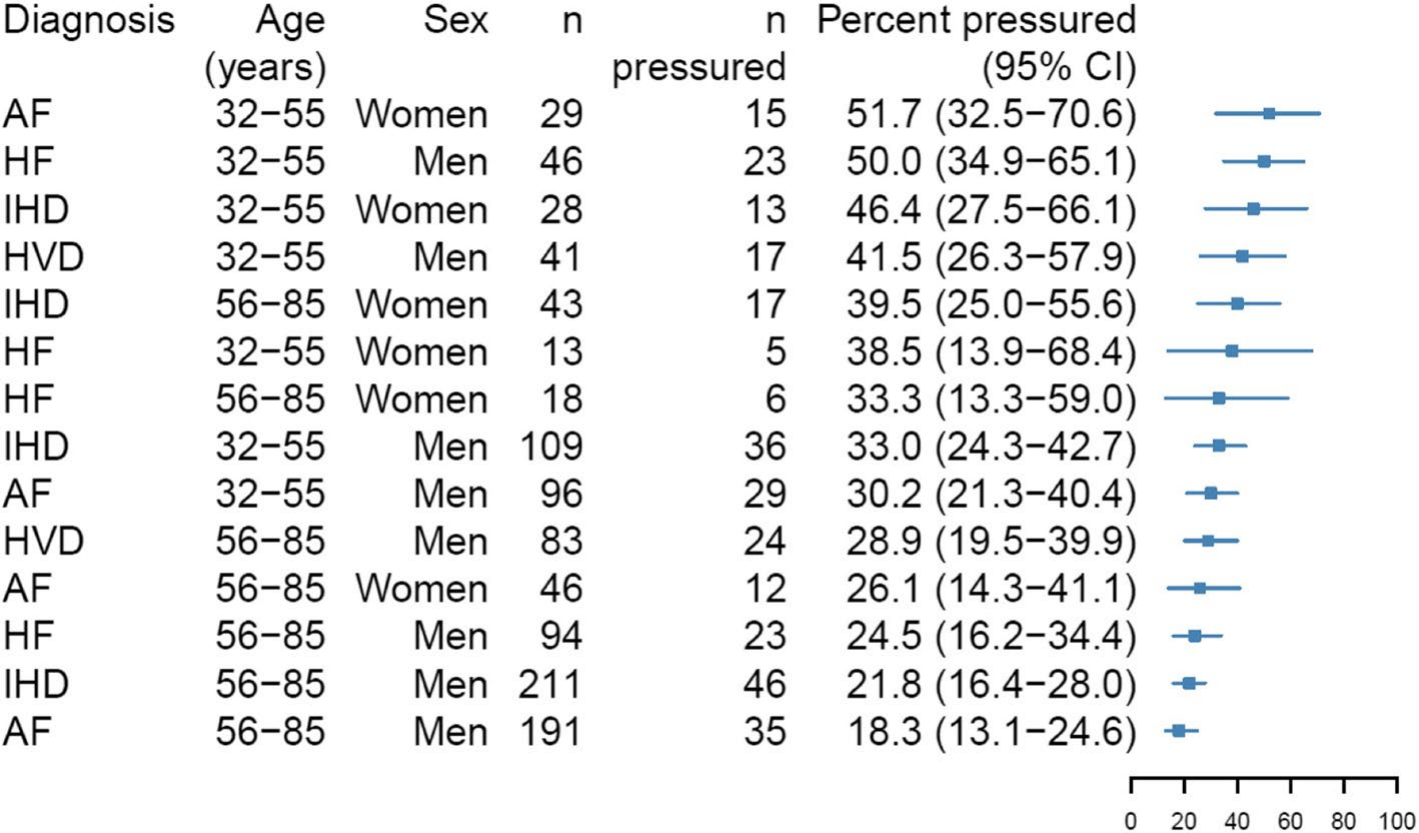
Probability of feeling under pressure to return to work for subgroups stratified by sex, age, and diagnosis. Probabilities are expressed as relative frequencies and reported with 95% confidence intervals (CI). Women with HVD were omitted as no pressure to return to work was reported by too few respondents. Abbreviations: AF, atrial fibrillation; HF, heart failure; HVD, heart valve disease; IHD, ischaemic heart disease

Figure 3 shows the probabilities of feeling under pressure to return to work (with 95% CIs) exhibited by subgroups stratified by diagnosis, age, and sex. The strata containing female respondents diagnosed with HVD were small, and because too few respondents had not felt pressured to return to work, these results are omitted to ensure anonymity. Almost all the women aged ≤ 55 years who had HVD reported pressure to return to work (data not shown). Among women aged ≤ 55 years with AF the observed probability of perceived return-to-work pressure was 51.7% (95% CI: 32.5–70.6), whereas the probability of pressure was 26.1% (95% CI: 14.3–41.1) for women aged > 55 years with AF. Among men, 50.0% (95% CI: 34.9–65.1) of respondents aged ≤ 55 years with HF reported pressure to return to work, while the probability of perceived pressure was 18.3% (95% CI: 13.1–24.6) among respondents aged > 55 years with AF.

## Discussion

In this nationwide combined survey- and register-based cohort study including people diagnosed with CVD during 2018, 842 respondents (53.8%) had returned to work when they answered the survey performed between October 2020 and February 2021. This return-to-work rate is lower than that reported by other studies [18–22], probably because our population had an older mean age than most previous studies. We investigated the likelihood of individuals feeling under pressure to return to work. The wording used to describe this pressure in Danish has negative connotations, and this was confirmed during our cognitive interviews. Almost one-third of respondents (29.7%) reported that they had felt under pressure to return to work, and we found that this experience was common across CVD diagnoses. Our results are probably most applicable to other welfare state nations, because patients’ economic and social-security safety nets, which affect the return to work [18], are shaped by the characteristics of each particular country. However, some patterns emerged that may inform the provision of cardiac rehabilitation programmes across western nations in general, improving the return-to-work experience.

First, our findings imply that returning to work may be more challenging for people diagnosed with HVD or HF than for those diagnosed with AF or IHD. This observation may be due to differing adverse effects on physical and mental health following CVD that are associated with each particular condition [31], and this finding is consistent with results from previous studies that reported higher return-to-work rates among people diagnosed with, for example, AF than HF [20, 22]. However, because the observed differences are modest, our findings suggest that vocational rehabilitation should not target particular patient groups, but rather focus on the individual’s ability to return to work and perform specific occupational tasks. The need for such an approach is demonstrated by the high prevalence of comorbidities (79.6%) and supported by a recent systematic review and meta-analysis that advocated focusing rehabilitation on each individual’s disease and condition [8].

Second, our findings indicate that people with CVD who are aged 55 years or younger are more likely to feel under pressure to return to work than people who are older than 55 years. This may be because younger people are less financially stable and more likely to have children living at home than older people. In addition, younger people may feel more susceptible to work-related factors. For example, employers may be more willing to retain senior employees who have more experience in that particular workplace. Furthermore, younger people may feel more pressure to return to work because they have more years left on the labour market and perhaps have higher career aspirations than people who are closer to retirement. Prolonged sickness absence is a major risk factor for permanent labour market exclusion [32], and returning to work following CVD may be especially important for younger people, who potentially have many years to work in the future. On the other hand, older people have a higher risk of permanent exclusion from the labour market due to ageism [33].

Third, our findings imply that women are more likely to feel under pressure to return to work than men. Regardless of their occupational status, women may take greater responsibilities for housework and caring duties at home [34], and this may increase their perceptions of pressure to return to work. In addition, more women than men reported a need for professional vocational counselling, and our findings indicated that women were more likely than men to return to work before feeling mentally and/or physically ready. This is a worrying observation and it is consistent with previous findings, which showed that many people with CVD, and particularly women, report a lack of psychosocial healthcare [35].

Overall, our study indicates that younger women may need special attention in cardiac rehabilitation, as suggested by previous studies [34]. Other authors have reported that women exhibit lower self-efficacy, lower quality of life, and greater psychological distress than men at the time of a first cardiac event or at the onset of rehabilitation [34, 36]. Furthermore, a recent review concluded that return to work is more strongly determined by psychosocial parameters than by the underlying cardiac disease [9], and there is strong evidence that anxiety and depression, which are common among people with CVD [37] have a negative impact on return to work [38]. Therefore, to facilitate a patient’s return to work all core rehabilitation components, including psychosocial support, must be considered. The value of combined programmes that include both exercise and counselling components, as well as individually delivered psychosocial and vocational interventions, has been demonstrated in recent reviews and meta-analyses [18, 39]. In addition, our results indicate that many people with CVD return to work before they feel ready. Therefore, vocational rehabilitation should, as suggested by other authors [19, 22], encompass employment maintenance to prevent reoccurrence of sick leave. Here, tele-rehabilitation may be a valuable approach, because this allows people to restart work while undergoing rehabilitation [9]. Moreover, to overcome some of the barriers to participation experienced by younger people (e.g., work responsibilities, time constraints and family responsibilities [40]), rehabilitation programmes need to be flexible and designed to suit the individual’s needs and preferences. Overall, tailored cardiac rehabilitation programmes are needed that reflect diversity in terms of disease severity and type, risk profile, age, sociocultural traditions, and everyday life situations [41].

In this study, the respondents indicated that job counselling centres were the most frequent source of pressure to return to work. In Denmark, all Danish citizens are entitled to paid sick leave, and job counselling centres are responsible for the administration of sickness absence. This finding is particularly relevant for the Nordic welfare state nations, but respondents also reported other sources of pressure (e.g., from their employer). This finding supports the creation of multidisciplinary and coordinated cardiac rehabilitation programmes that encourage relevant stakeholders (e.g., employers, social workers, rehabilitation nurses and physiotherapists) to develop individualized return-to-work plans. Such an approach may hold potential for improving vocational rehabilitation across Europe, where many countries currently lack clear guidelines [9].

Finally, in addition to feeling both pressured and unready to return to work, we found that half of our respondents who expressed a need for professional vocational counselling reported that these needs were unmet. Therefore, similar to previous European studies [9, 42], our results imply that current vocational cardiac rehabilitation programmes are inadequate and that a greater focus on this component is warranted.

### Strengths and limitations

We invited 10,000 people with CVD to participate in the Life With Heart Disease study. Random selection from the cohort of all patients who had a hospital admission (in- or outpatient contact) in 2018 with a discharge diagnosis of one of the four most common CVD generated a representative database. All respondents were diagnosed with at least one of four CVD in 2018, and the study provided insight into the experiences of a large and relatively homogenous group of CVD patients. In addition, we used recommended practices to develop our questionnaire including pilot testing, which increases the validity of the data.

Our study had some limitations. Although we used a valid register to generate our cohort [43], not all individuals identified in the Life With Heart Disease study had the CVD that was registered. In The Danish National Patient Register, the positive predictive value for CVD diagnoses is 88% [43]. Therefore, to take this into account, we included self-reported CVD diagnoses in the survey and excluded people who stated that they were not diagnosed with CVD in 2018.

The total response rate was lower than expected, which is likely due to the length of the questionnaire (the mean answering time was 59 min), difficulties contacting some respondents due to incorrect phone numbers, and the reminder calls being sent from an anonymous number. Furthermore, our questionnaire was sent out during the COVID-19 pandemic.

The relatively small study population limited the statistical power of the study, and non-response bias should be considered. Non-respondents had a lower educational level and a lower mean income than respondents. Because these factors have previously been associated with failure to return to work and reoccurrence of sick leave [19, 22], the prevalence of pressure to return to work may have been underestimated. Individuals who have a lower socioeconomic status may be under more pressure to return to work than those who have a higher socioeconomic status because the former are more likely to be underinsured and have less favourable sick leave arrangements and short-term disability benefits [44]. Moreover, the pressure to return to work may be even greater in countries with fewer welfare benefits and more societal and economic inequalities.

The risk of recall bias should also be considered because respondents answered the questionnaire between 1.75 and 3 years after their diagnosis. The respondents’ recollections of their experiences when returning to work may have been influenced by their working situation at the time when they answered the survey. Respondents who were satisfied with their current working conditions may have forgotten whether they had previously felt under pressure to return to work. However, questions regarding return to work were carefully formulated and informants had no difficulty remembering their return-to-work experiences during cognitive interviews. Such experiences are often memorable, minimizing the risk of recall bias [45]. In addition, as participants answered the survey during the COVID-19 pandemic, it should be mentioned that we do not expect that this has affected perceived return-to-work pressure. As all participants answered the survey more than 1.5 years after their CVD diagnosis, we believe that the majority had returned to work before the onset of the pandemic.

Our study only covered the four most common CVD diagnoses, and only included people who were 32 years or older. Younger people, and people with other forms of CVD may experience different challenges when returning to work. In addition, we only included respondents who had actually returned to work. Therefore, future studies should investigate the needs and barriers to returning to work experienced by patients with CVD who remain on sick leave. In this regard, qualitative studies are needed to unfold patients’ perspectives on return to work and to gain a deeper understanding about how return-to-work pressure takes place and is experienced.

Finally, we could have collected valuable information regarding work-related factors, such as occupational requirements (e.g., job function and tasks) that influence the return to work [18]. In addition, most of our respondents were of Danish origin. It is likely that the return-to-work experience of non-Danish speaking people varies from that of our Danish respondents; other authors have found language-related barriers to participate in cardiac rehabilitation and inadequate provision of core components of cardiac rehabilitation for non-Danish speaking people [46].

## Conclusions

In this nationwide combined survey- and register-based study, almost one-third of the study population reported that they had felt under pressure to return to work. In addition, our findings indicated that many people with CVD return to work before they feel physically or mentally ready, and that their needs for vocational rehabilitation are not being met. Pressure to return to work was associated with age, sex, and CVD diagnosis, and our results suggest that more flexible, person-centred return-to-work interventions that can meet diverse needs are required for this patient group. In addition, our study emphasizes the value of multidisciplinary and coordinated interventions that involve relevant stakeholders, as well as the importance of ensuring that cardiac rehabilitation programmes include all core rehabilitation components, including both psychosocial support and vocational rehabilitation.

## Data Availability

The data that support the findings of this study are not publicly available due to The Danish legislation on data security. Data are however available from the authors upon reasonable request.

## Abbreviations

CVD: Cardiovascular disease
CI: Confidence interval
ICD-10: International Classification of Diseases 10th Revision
AF: Atrial fibrillation
IHD: Ischaemic heart disease
HVD: Heart valve disease
HF: Heart failure
